# The Involvement of Aβ42 and Tau in Nucleolar and Protein Synthesis Machinery Dysfunction

**DOI:** 10.3389/fncel.2018.00220

**Published:** 2018-08-03

**Authors:** Mahmoud B. Maina, Laura J. Bailey, Aidan J. Doherty, Louise C. Serpell

**Affiliations:** ^1^School of Life Sciences, University of Sussex, Brighton, United Kingdom; ^2^Department of Human Anatomy, College of Medical Sciences, Gombe State University, Gombe, Nigeria; ^3^Genome Damage and Stability Centre, School of Life Sciences, University of Sussex, Brighton, United Kingdom

**Keywords:** Aβ42, oxidative stress, rDNA transcription, rRNA processing, RNA synthesis, protein synthesis, Alzheimer’s disease, Tau

## Abstract

Alzheimer’s disease (AD) is the most common form of dementia and is distinguished from other dementias by observation of extracellular Amyloid-β (Aβ) plaques and intracellular neurofibrillary tangles, comprised of fibrils of Aβ and tau protein, respectively. At early stages, AD is characterized by minimal neurodegeneration, oxidative stress, nucleolar stress, and altered protein synthesis machinery. It is generally believed that Aβ oligomers are the neurotoxic species and their levels in the AD brain correlate with the severity of dementia suggesting that they play a critical role in the pathogenesis of the disease. Here, we show that the incubation of differentiated human neuroblastoma cells (SHSY5Y) with freshly prepared Aβ42 oligomers initially resulted in oxidative stress and subtle nucleolar stress in the absence of DNA damage or cell death. The presence of exogenous Aβ oligomers resulted in altered nuclear tau levels as well as phosphorylation state, leading to altered distribution of nucleolar tau associated with nucleolar stress. These markers of cellular dysfunction worsen over time alongside a reduction in ribosomal RNA synthesis and processing, a decrease in global level of newly synthesized RNA and reduced protein synthesis. The interplay between Aβ and tau in AD remains intriguing and Aβ toxicity has been linked to tau phosphorylation and changes in localization. These findings provide evidence for the involvement of Aβ42 effects on nucleolar tau and protein synthesis machinery dysfunction in cultured cells. Protein synthesis dysfunction is observed in mild cognitive impairment and early AD in the absence of significant neuronal death.

## Introduction

Alzheimer’s disease (AD) is associated with memory impairment and is characterized by the deposition of extracellular amyloid plaques and intracellular neurofibrillary tangles. Amyloid-β (Aβ42), the main constituent of the plaques, is believed to be the most toxic species that drives the pathogenesis of AD according to the original amyloid cascade hypothesis (Hardy and Higgins, 1992). This is supported by biomarker studies, which show that changes in Aβ appear decades before the onset of dementia (Jack et al., 2013). The reformulated amyloid cascade hypothesis focuses on soluble, oligomeric Aβ species as the mediators of neuronal toxicity that may be associated with downstream effects on tau (Selkoe and Hardy, 2016). Levels of Aβ oligomers have been shown to correlate with the disease severity (DaRocha-Souto et al., 2011), while distribution of tau neurofibrillary tangles correlates with disease progression (Braak and Braak, 1991). Aβ42 oligomers have been found to cause synaptic aberration (Lacor et al., 2007), disrupt long-term potentiation (LTP) (Lambert et al., 1998), and cause memory dysfunction (Zhang et al., 2014), indicating that the Aβ42 oligomers cause a gradual disturbance in cell function before neuronal loss. Whether Aβ changes impact on tau remains unclear.

Protein synthesis is known to be necessary for formation and stability of long-term memory. The synthesis of new proteins is a regulated process that is coordinated from the nucleolus to the ribosomes. This begins with the formation of functional ribosomes, which consist of 18S and 28S ribosomal RNA (rRNA), derived from 45S-pre-ribosomal RNA (45S-pre-rRNA). Several studies have identified nucleolar stress, ribosome dysfunction, and alteration of protein synthesis machinery as neurochemical changes that accompany early AD (Ding et al., 2005; Hernandez-Ortega et al., 2015). Ribosomal dysfunction, decreased levels of rRNA and tRNA, and reduced protein synthesis, have been observed in the brains of patients with mild cognitive impairment (MCI), indicating that these changes precede neuronal loss that occurs during AD (Ding et al., 2005). A decrease in many nucleolar proteins and their mRNA have also been reported in early AD (Hernandez-Ortega et al., 2015), a period associated with less neuronal loss (Braak and Braak, 1991). This seems to suggest that the protein synthesis machinery becomes altered before the onset of AD and this progresses with the disease process. Interestingly, tau has been shown to play a role in the nucleus and nucleolus as well as being a microtubule binding protein in the cytosol (Maina et al., 2018).

We have previously shown that Aβ42 oligomers enter undifferentiated neuroblastoma (SHSY5Y) cells leading to lysosomal damage (Soura et al., 2012). In primary hippocampal neurons, we showed that Aβ42, but not a non-toxic variant, becomes internalized and alters synaptic vesicle recycling properties (Marshall et al., 2016), establishing a critical role for Aβ42 in driving the neurochemical changes in AD. Here, we investigated the hypothesis that Aβ42 could impact on localization and function of tau and may be important in the impairment of the protein synthesis machinery observed in early AD. We reveal that incubation of differentiated SHSY5Y cells with Aβ42 oligomers leads to increased oxidative stress and a gradual accumulation of nucleolar stress. These changes result in altered production and processing of 45S-pre-ribosomal rRNA (45S-pre-rRNA), heterochromatin compaction and a decrease in RNA and protein synthesis, without DNA damage or loss of cell viability. Furthermore, Aβ42 oligomer administration results in changes in localization and altered phosphorylation state of nuclear human tau protein in differentiated SHSY5Y cells. Changes in levels of nuclear phosphor-tau have been observed with AD progression (Hernandez-Ortega et al., 2015). This is the first observation of a central involvement of Aβ42 in nucleolar dysfunction and altered protein synthesis machinery in a cellular model, which recapitulates the scenario that occurs from MCI to AD (Ding et al., 2005; Hernandez-Ortega et al., 2015). This demonstrates that the early changes in Aβ42 levels that occur decades before full-blown AD (Jack et al., 2013) may influence nuclear tau, and contribute to the altered protein synthesis machinery, from the nucleolus to the ribosome, which occurs at the early stage of the disease.

## Materials and Methods

### Cell Culture

Undifferentiated SHSY5Y neuroblastoma cells (Sigma-Aldrich and originally supplied by Public Health England culture collections) were maintained in Dulbecco’s Modified Eagle Medium: Nutrient Mixture F-12 (DMEM/F-12) (Life Technologies, United Kingdom), supplemented with 1% (v/v) L-glutamate (L-Gln) (Invitrogen), 1% (v/v) penicillin/streptomycin (Pen/Strep) (Invitrogen) and 10% (v/v) Fetal Calf Serum (FCS) at 37°C and 5% CO_2_. SHSY5Y cells were differentiated in a medium containing 1% FCS supplemented with 10 μM trans-Retinoic acid (Abcam) for 5 days. On day five, the medium was replaced with a serum-free media supplemented with 2 nM brain-derived neurotrophic factor (BDNF) (Merck Millipore). Cells were used 2 days post-BDNF incubation.

### Preparation of Aβ

Aβ42 (rPEPTIDE) was prepared following an established procedure (Soura et al., 2012). Briefly, the peptide was solubilized in 1,1,1,3,3,3-hexafluoro-2-propanol (99% purity) (Fluka, Sigma-Aldrich) and sonicated. The HFIP was removed and dried Aβ was dissolved in Dimethyl sulfoxide (DMSO) >99.9% (ACROS Organics) at 0.2 mg/ml. Solvents were removed using a 5 ml HiTrap desalting column (GE Healthcare) in 30 μL of Hepes buffer [10 mM Hepes, 50 mM NaCl, 1.6 mM KCl, 2 mM MgCl_2_⋅6H_2_O, 3.5 mM CaCl_2_⋅2H_2_O (pH 7.4)]. Aβ peptide concentration was determined using a Nanodrop spectrophotometer (Thermo Fisher Scientific) at a wavelength of 280 nm (extinction coefficient of 1490). An equivalent of 10 μM from the Aβ42 stock was administered to the medium of the differentiated SH-SY5Y cells.

### Western Blotting

SHSY5Y cells were fractionated for 15 min on ice using RIPA (Abcam), supplemented with protease (Sigma) and phosphatase (Sigma) inhibitors and spun at 16000 ×*g* for 15 at 4°C. Protein concentration was quantified using Pierce BCA Protein Assay Kit (Thermo Fisher Scientific) and absorbance (562 nm) was read using GloMax Multi-Detection plate reader (Promega). A total of 10 μg of protein from each sample was diluted in 4× Laemmli sample buffer, supplemented with 1:10 dilution of β-mercaptoethanol, then loaded to 7.5% Mini-PROTEAN TGX Stain-Free Protein Gels (BIO-RAD) and SDS-PAGE was run at 100V using 1× running buffer (25 mM Tris, 192 mM glycine, 0.1% SDS). The proteins were transferred to PVDF membrane (Merck Millipore) using 1× transfer buffer [25 mM Tris–HCl, 192 mM glycine, and 10% (v/v) methanol] at 100 V. The membrane was blocked in 5% (w/v) milk dissolved in washing buffer (TBS-Tween) (Merck Millipore), incubated at 4°C overnight with primary antibodies diluted in the blocking buffer (see Supplementary Table 1 for antibodies). The membranes were washed 5× for 10 min and probed for 1 h with corresponding secondary antibodies diluted in blocking buffer. The membranes were washed 5× for 10 min each and subsequently developed in the dark room after incubation in Clarity Western ECL substrate for 1 min (BIO-RAD). To ensure the specificity of the secondary antibodies, control experiments were run using secondary antibodies, without primary antibodies, and this did not show any specific chemiluminescent signal. For loading control antibodies or sequential analyses of other proteins on the same membrane using other antibodies, the membranes were stripped using Restore^TM^ PLUS Western Blot Stripping Buffer (Thermo Fisher Scientific), then blocked, and probed as described above. The blots were scanned at high resolution, and then bands were quantified using ImageJ software.

### Immunofluorescence Labeling

SHSY5Y cells were resuspended in PBS and spun onto a glass slide at 800 RPM for 3 min using Cytospin Centrifuge (CellSpin I, Tharmac). Cells were fixed with 4% paraformaldehyde/PBS for 15 min, PBS-washed, permeabilized using 0.5% Triton X-100/PBS for 15 min and PBS-washed. The slides were blocked in blocking buffer [4% BSA/PBS/Tween-20 (0.02%)] for 45 min, incubated with primary antibody diluted in the blocking buffer for 45 min, PBS-washed, incubated in Alexa fluorophore-conjugated corresponding secondary antibody diluted in the blocking buffer for 45 min. The slides were PBS-washed, incubated in 1/1000 DRAQ5 (Abcam) diluted in PBS/Tween-20 (0.02%) for 10 min and mounted with coverslips using ProLong^®^ Gold Antifade mountant (Life Technologies). 5-Methylcytosine (5-mC), cells on the glass slides were fixed with 2.5% PFA/PBS for 30 min at RT, PBS-washed, permeabilized for 1 h at RT with 0.5% Triton X-100/PBS. The cells were then washed in wash buffer [PBS/0.1% Triton X-100 (PBST)] and incubated with 2N HCl for 30 min at 37°C to depurinate the DNA, followed by 2× 5 min wash with 0.1 M borate buffer (pH 8.5). They were then rinsed twice with PBS-T, blocked in blocking buffer (1% BSA/PBS-T) for 1 h at RT, incubated with the primary antibody diluted in the blocking buffer for 2 h at RT and washed three times with PBS-T. Then they were incubated with the corresponding secondary antibody diluted in the blocking buffer for 45 min at RT in the dark and washed three times in PBS-T, then stained with DRAQ5.

### Confocal Microscopy Imaging and Analysis

Images were taken using a 100× oil objective of LSM510 Meta confocal microscope mounted on Axiovert200M using pinhole size of 1 Airy unit. All images were collected as Z-stacks for all channels using a step size of 1 μm to allow the analysis of the entire signal in the cells. Subsequently, images were Z-projected to sum all signals and then analyzed using ImageJ. Five randomly collected images from each experiment and an average of 50–70 cells per experiment were subjected to the ImageJ analysis. For the quantification of nuclear foci/cluster, ImageJ Procedure presented by the light microscopy core facility of Duke University was used. For the quantification of total nuclear fluorescence intensities, the nuclei were first segmented by thresholding using the DRAQ5 channel, excluding fused nuclei or those at the edges. Subsequently, the multi-measure option on the ImageJ ROI manager was used to measure nuclear fluorescence from all channels in only segmented nuclei. The total corrected nuclear fluorescence (TCNF) was then calculated as TCNF = Integrated Density – (Area of selected cell × Mean fluorescence of background readings) (McCloy et al., 2014). For the quantification of nucleolar nP-Tau, a similar approach was used, in which the nucleolus was first segmented by thresholding using the fibrillarin channel. For the quantification of nucleolar nP-Tau and FBL redistribution, Z-stack images were Z-projected to maximum intensity before cells positive for the redistribution were counted.

### CellROX Green Assay

SHSY5Y cells were incubated with 5 μM CellROX Green Reagent for 1 h at 37°C and 5% CO_2_ (Life Technologies, United Kingdom). The cells were resuspended in PBS and analyzed on a FACS using the 488 nm excitation laser (BD Accuri C6, BD Biosciences). A total of 10,000 events were collected per sample and resulting FL1 data were plotted on a histogram.

### Nascent RNA and Protein Synthesis

Nascent RNA and protein synthesis were visualized, respectively, using Click-iT RNA Alexa Fluor 488 Imaging Kit (Life Technologies) and Click-iT HPG Alexa Fluor 488 Protein Synthesis Assay Kit (Life Technologies) following the manufacturer’s instructions and images were taken using a 100× oil objective of LSM510 Meta confocal microscope mounted on Axiovert200M using pinhole size of 1 AU.

### RNA Extraction and Complementary DNA (cDNA) Synthesis

Total RNA was extracted using TRIzol Plus RNA Purification Kit (Life Technologies, United Kingdom). SHSY5Y were lysed directly with 1 mL TRIzol reagent for 5 min at RT. The lysates were resuspended and transferred to separate 1.5 mL tubes, mixed with 200 μL chloroform, agitated by hand vigorously for 15 s, and incubated for 2–3 min at RT. The samples were spun at 12000 ×*g* for 15 min at 4°C and the upper aqueous phase which contains the RNA was collected. About 450 μL of the top aqueous phase from each sample was transferred to new RNase-free tubes and mixed vigorously with an equal volume of 70% ethanol to obtain 35% ethanol in the mixture and these were transferred to separate spin cartridges (with a collection tube), spun at 12,000 ×*g* for 15 s at RT and the flow through was discarded. The cartridges were washed with buffer I, spun at 12,000 ×*g* for 15 s at RT and further washed twice with buffer II at 12,000 ×*g* for 15 s at RT. The cartridges containing the RNA were dried by additional spin at 12,000 ×*g* for 1 min at RT. Using recovery tubes, the RNA from the different cartridges was eluted after a 5 min incubation in 30 μL RNase-free water and spun for 2 min at 16000 ×*g*. The RNA extracts were stored on ice and used for cDNA synthesis. The total RNA extracted was used for cDNA synthesis using the High Capacity cDNA Reverse Transcription Kit (Life Technologies, United Kingdom). A 20 μL cDNA reaction was prepared for each sample on ice, in PCR tubes, containing 10 μL total RNA and 10 μL 2× Reverse Transcription master mix supplemented with RiboLock RNase Inhibitor at a concentration of 1 U/μL of a reaction mixture (Life Technologies). All tubes were briefly spun to eliminate bubbles and loaded to the thermal cycler (Biometra), programmed to run at 25°C for 10 min, 37°C for 120 min and 85°C for 5 min. The cDNA was collected and used for qPCR.

### Quantitative Polymerase Chain Reaction (qPCR)

The synthesized cDNA from all samples were subjected to qPCR using Maxima Probe/ROX qPCR Master Mix (2×) Kit (Life Technologies) and Taqman gene expression assays (Life Technologies, United Kingdom, Supplementary Table 2). A 1× master mix sufficient for a 25 μL-reaction for all samples in duplicates was prepared from the Maxima Probe/ROX qPCR Master Mix (2×), 20× TaqMan gene expression assay and Nuclease-free water, and 20 μL of the mixture were transferred to required wells of a white 96-well semi-skirted PCR plate for Roche Lightcycler (StarLab, United Kingdom). A cDNA serial dilution of 1:1, 1:10, 1:100, and 1:1000 was prepared for standard curve measurement and 5 μL of cDNA samples were transferred to corresponding wells of the 96-well PCR plate. A fresh master mix and standard curve were prepared for each assay and template negative controls containing only nuclease free H_2_O were included in every amplification. Absolute qPCR was carried out on all samples using Roche LightCycler 480 II (Roche Diagnostics, Basel, Switzerland). The cycling conditions used were an initial run at 50°C for 2 min, initial denaturation at 95°C for 10 min, and 50 cycles of denaturation at 95°C for 15 s and annealing and extension at 60°C for 1 min and finally cooling at 4°C. After the qPCR, transcript levels were automatically determined using the standard curve method by the Roche LightCycler 480 service software. Samples were normalized to TBP and ACTB.

### Statistical Analysis

All data were subsequently subjected to *Kolmogorov–Smirnov (K-S)* normality test and then unpaired student’s *t*-test using GraphPad InStat.

## Results

### Aβ42 Induces Oxidative Stress and Alters Tau Phosphorylation and Localization Without Cell Viability Loss or DNA Damage

**FIGURE 1:**
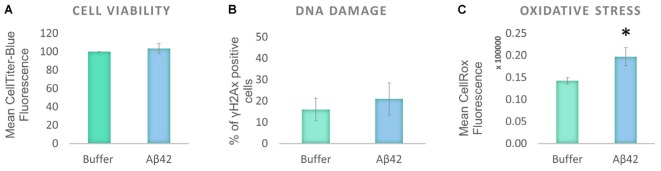
Aβ42 oligomers induce oxidative stress, without DNA damage or loss of cell viability 24 h post-incubation. **(A)** CellTiter Blue experiment showed that the Aβ incubation had no effect on cell viability. **(B)** Quantification of γ-H2Ax-positive cells showed the absence of DNA damage following the Aβ incubation. **(C)** Flow Cytometry Experiment with CellROX Green showed that the Aβ induces a significant level of oxidative stress. *N* = 5. ^∗^*P* < 0.05.

To investigate the effect of oligomeric Aβ on human tau distribution, a human neuroblastoma cell line, SHSY5Y, was selected as it produces normal endogenous levels of human tau. This contrasts with mouse or rat tau produced in primary neurons from laboratory animals, or with over-expression of human tau from transgenic animal cells and provides a model cellular system with which to explore human tau at normal levels. The cells were differentiated to increase their neuronal characteristics (henceforth called D.SHSY5Y) and treated with freshly prepared Aβ42 oligomers (10 μM). Viability of the differentiated cells was unaffected after 24 h incubation with oligomeric Aβ42 oligomers (**Figure 1A**). This is in contrast to previous studies that show that undifferentiated SHSY5Y cell viability is reduced by Aβ42 oligomers (Soura et al., 2012). To investigate whether more subtle dysfunctional effects were induced in differentiated cells, we examined whether the Aβ causes DNA damage using a well-known DNA damage marker, γH2Ax foci (Podhorecka et al., 2010). The Aβ-treated D.SHSY5Y showed no significant increase in numbers of γH2Ax foci (**Figure 1B**). Aβ has been suggested to lead to oxidative stress (Butterfield et al., 2013) so we explored this in our cellular system using the CellROX Green flow cytometry assay. This revealed a significant increase in oxidative stress (13% increase) in the D.SHS5Y5 cells (**Figure 1C**), suggesting that Aβ42 can cause oxidative stress, without exerting significant cell viability loss or DNA damage on these differentiated cells over the incubation period of 24 h.

**FIGURE 2:**
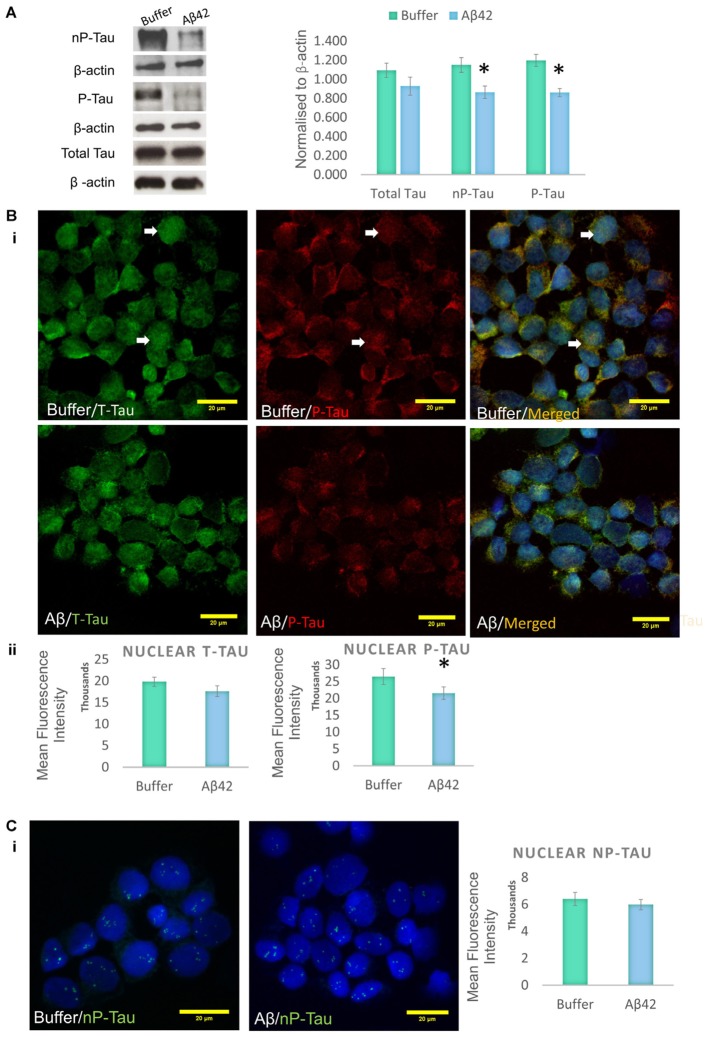
Aβ42 oligomers change the phosphorylation of tau epitopes. **(A)** Western blotting on whole cell extracts showing the levels of Tau Thr231 (P-Tau), Tau-1 (nP-Tau) and total tau (T-Tau) following Aβ administration. Normalized to β-actin. Immunofluorescence labeling indicating the presence of nuclear phosphorylated tau **(Bi)**, which significantly decreases following the Aβ treatment, without changes in total nuclear tau **(Bii)** or nuclear nP-Tau **(C)**. *N* = 5. ^∗^*P* < 0.05.

Aβ toxicity is widely believed to have an impact on tau protein, and several studies suggest that tau modifications, such as phosphorylation/dephosphorylation, may be a signature of general cellular stress (Egaña et al., 2003; Galas et al., 2006; Kátai et al., 2016). It has previously been shown that the differentiation protocol used increases tau phosphorylation in SHSY5Y cells, making the model ideal for the investigation of AD-like changes (Jamsa et al., 2004). Western blotting was used to investigate whether the Aβ treatment impacts on tau phosphorylation in D.SHSY5Y cells (whole cells). The levels of two major tau species were measured and these were compared to levels of total tau (henceforth referred to as T-Tau): (1) Tau phosphorylated on Thr231 is one of the key sites phosphorylated in AD, occurring before structural changes to the tau molecule (Luna-Munoz et al., 2005) (henceforth referred to as P-Tau) and; (2) Tau dephosphorylated on Ser 195, 198, 199, and 202 using Tau-1 antibody (henceforth referred to as nP-tau). Cells incubated with oligomeric Aβ42 show a significant decrease in P-Tau and nP-Tau (**Figure 2A** and Supplementary Figure S2). However, changes to whole cell T-tau levels compared to cells treated with buffer alone were not significant (**Figure 2A**). A decrease in nP-Tau indicates an increase in phosphorylation at positions Ser 195, 198, 199, and 202. Considering the several epitopes on the tau molecule that can be post-translationally modified, these changes suggest a dynamic phosphorylation of different epitopes of tau following Aβ induced stress.

**FIGURE 3:**
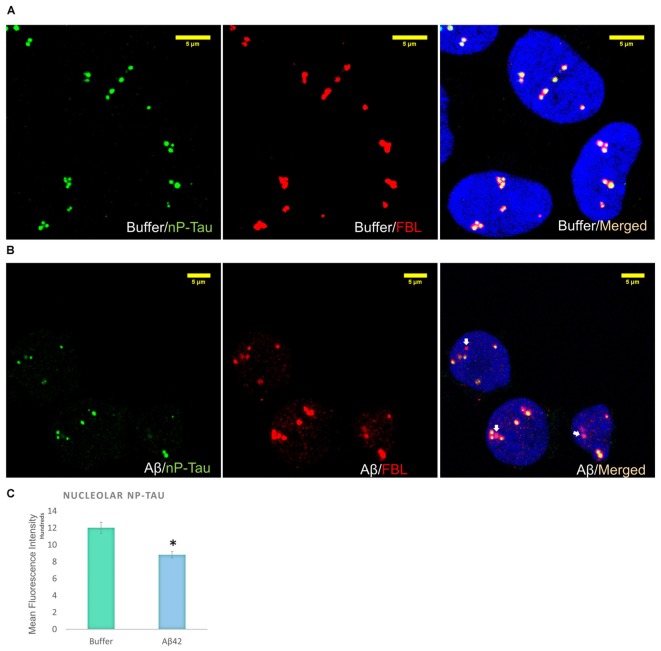
Aβ42 oligomers alter nucleolar tau localization 24 h post-incubation. Immunofluorescence labeling in buffer-treated cells show co-localization between nP-Tau and FBL **(A).** 10 μM Aβ42 treatment induces the delocalization of nP-Tau from FBL **(B).** Quantification showed that this altered colocalization is associated with a significant reduction in the nucleolar nP-Tau **(C)** *N* = 5. ^∗^*P* < 0.05.

Incubation of D.SHSY5Y with Aβ42 for 8 h (Noel et al., 2016) or N2a cells with formaldehyde for 2–4 h (Lu et al., 2013b), have been shown to result in the accumulation of phosphorylated tau in the nucleus. Therefore, we focussed on the effect of Aβ oligomer incubation for 24 h on the nuclear tau species. For this, we employed immunofluorescence imaging by the collection of Z Stacks to enable direct visualization of the nuclear localized tau with DRAQ5 co-fluorescence and allow unbiased quantification of the signals through the entire nuclear volume (**Figure 2B**). Immunofluorescence suggested no change in T-Tau following Aβ42 incubation compared to buffer treated control cells (**Figure 2B**) and comparison of nuclear levels of T-Tau were not statistically significantly different (**Figure 2B**). Buffer-treated D.SHSY5Y showed immunolabeling of P-Tau in the nucleus (**Figure 2B**) and the level of P-tau decreased significantly following Aβ42 incubation (**Figure 2B**), consistent with the Western blotting results showing reduction of P-Tau in the whole cell following Aβ42 incubation (**Figure 2A**). Importantly, merged images labeled for T-Tau and P-Tau reveal an amount of T-Tau that is not detected by the P-tau antibody, so we explored whether this represented nP-Tau. Depending on the immunofluorescence detection protocol used (Lu et al., 2014), nP-Tau appears as small, discrete, punctate fluorescence within the nucleus in contrast to T-Tau and P-Tau which show more diffuse staining (**Figure 2C**). Nuclear levels of nP-Tau in Aβ42 and buffer treated cells were quantified and compared and revealed no significant change following Aβ42 incubation (**Figure 2C**), though there was a small but significant decrease in nP-Tau levels in the total cell extract revealed by Western blotting (**Figure 2A**). Fibrillarin (FBL) is a marker for nucleoli and nuclear nP-Tau shows distinct punctate labeling that co-localizes with the nucleolar protein, FBL in untreated cells (**Figure 3A**). To investigate the impact of Aβ incubation on this nucleolar nP-Tau, we used FBL fluorescence to segment the nucleolus, then quantified the abundance of nP-Tau fluorescence following Aβ incubation. This showed a significant reduction in the nucleolar-nP-Tau (**Figures 3B,C**). Since we found no difference in the total nuclear levels of nP-Tau (**Figure 2Ci**), the decrease in the nucleolar-specific nP-Tau suggests changes in its nuclear/nucleolar ratio, which has also been reported for other nucleolar proteins like FBL (Kodiha et al., 2011). Overall, these results showed that the Aβ incubation leads to a reduction in total cell P-Tau and nP-Tau levels and specifically decreases the levels of nucleolar nP-Tau. Close inspection of the distribution of nucleolar marker FBL in Aβ42 treated cells reveals the expected puncta but also very small speckles which may signal some nucleolar distress.

### Aβ42 Induces Nucleolar Stress and Reduces RNA and Protein Synthesis Levels

**FIGURE 4:**
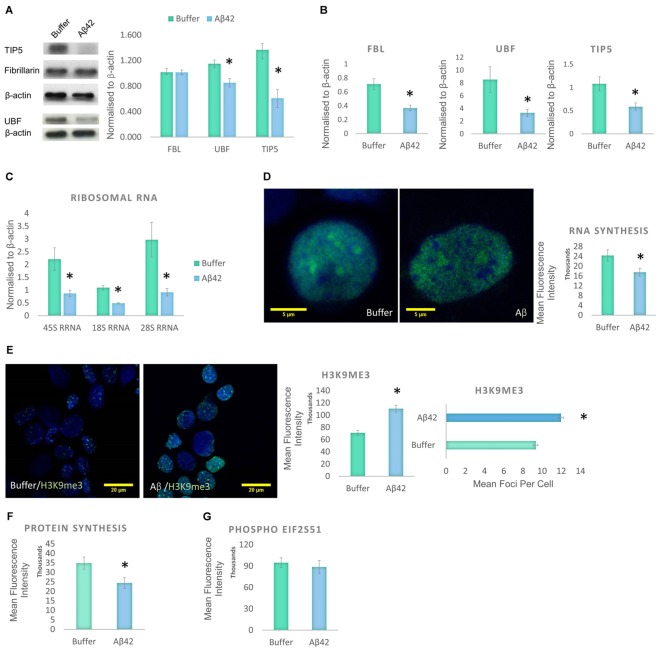
Aβ42 induces nucleolar stress and inhibit RNA and Protein Synthesis 24 h post-incubation. **(A)** Western blotting revealed that Aβ treatment led to a significant decrease in UBF, TIP5, but not FBL. Normalized to β-actin. **(B)** qPCR analysis showed a significant reduction of UBF, TIP5, and FBL transcripts. Normalized to β-actin or TBP. **(C)** qPCR analysis of rDNA transcription and processing showed that the Aβ incubation resulted in a significant decrease in 45S pre-rRNA synthesis and processing of 18S rRNA and 28S rRNA. Normalized to β-actin or TBP. **(D)** Quantitative Click-iT RNA immunofluorescence labeling showed that the Aβ causes a global reduction in newly synthesized RNA, **(E)** which is associated with a nuclear increase in H3K9me3 intensity and foci. **(F)** Quantitative Click-iT HPG Alexa Fluor 488 immunofluorescence labeling showed a significant decrease in nascent protein synthesis following the Aβ treatment. **(G)** Quantitative immunofluorescence labeling for phosphor S51 eukaryotic translation initiation factor 2A (EIF2A-P) showed no changes following the Aβ treatment. For **A**, *N* = 5, **B–E**, *N* = 4 and **F,G**, *N* = 3, ^∗^*P* < 0.05 (See also Supplementary Figure 1).

Cellular stress is known to disrupt the integrity of the nucleolus (Boulon et al., 2010). Therefore, the decrease in nucleolar nP-Tau prompted us to investigate whether the nucleolus was under stress. Stress can cause the nucleolus to undergo reorganization associated with the degradation and redistribution of nucleolar proteins. To investigate the presence of nucleolar stress, we quantified the levels of UBF, TIP5 and FBL using western blotting of the whole cell extract. UBF is a nucleolar transcription factor that drives the transcription of rDNA (Sanij et al., 2008; Bártová et al., 2010). TIP5 is a member of the nucleolar remodelling complex (NoRC) that mediates the silencing of a fraction of rDNA, leading to heterochromatin formation and transcriptional silencing (Santoro et al., 2002). Aβ42 incubation led to a significant decrease in the levels of UBF and TIP5 but no changes in FBL levels (**Figure 4A**). To examine whether these changes occur specifically at the protein production or transcript level, we quantified their respective gene expression levels using qPCR. Aβ42 incubation resulted in a significant decrease in the gene expression levels of FBL, UBF, and TIP5 (**Figure 4B**). This may indicate a reduction in transcription as a likely contributor to the reduction observed at the protein level. The absence of a difference in the protein level of FBL (**Figure 4A**), despite the decrease in its transcript, may be due to a longer half-life of the protein (Vogel and Marcotte, 2012). To further confirm the presence of nucleolar stress, we examined whether the Aβ42 incubation affects rDNA transcription and processing. The reduction in transcription or maturation of 28S and 18S rRNA or increase in their degradation has been suggested to contribute to nucleolar dysfunction in AD (da Silva et al., 2000). qPCR analysis showed that the Aβ led to a significant reduction of 45S pre-rRNA and levels of 18S and 28S rRNA (**Figure 4C**). Thus, these findings reaffirm that Aβ incubation results in nucleolar stress with an associated reduction of rRNA production and processing.

Given the changes observed in the levels of 45S-pre-rRNA and gene expression levels of other nucleolar proteins, global RNA synthesis levels were examined using the Click-iT RNA Imaging assay. Incubation of D.SHS5Y5 cells with oligomeric Aβ42 showed a significant reduction in nascent RNA synthesis (**Figure 4D**). To explore whether these changes may be due to changes in heterochromatin levels, H3K9me3 was used as a constitutive heterochromatin marker. H3K9me3 is known for its role in transcriptional repression, and it accumulates to form foci at constitutive heterochromatin (Saksouk et al., 2015). A significant increase was observed in the nuclear level of H3K9me3 and its foci in D.SHSY5Y cells incubated with Aβ42 (**Figure 4E**). This finding compliments the qPCR and Click-iT RNA labeling data, which reveal a decline in RNA levels (**Figures 4B,D**). Since we observed a global increase in heterochromatin, a decrease in RNA synthesis and rDNA transcription, we explored whether this culminates in a reduction in synthesized proteins. Using Click-iT HPG Protein Synthesis Assay, we observed that incubation of cells with Aβ led to a significant decrease in the global levels of newly manufactured proteins (**Figure 4F**). Phosphorylation of elFα plays an important role in regulation of protein synthesis during homeostatic control and stress (Holcik and Sonenberg, 2005). An increased level of eIF2α phosphorylation has been associated with the pathogenesis of AD (Hoozemans et al., 2009), as well as other neurodegenerative diseases (Halliday and Mallucci, 2015). Therefore, we next investigated whether Aβ incubation also affects the eIF2α pathway. However, using quantitative eIF2α immunofluorescence and western blotting, no difference in eIF2α phosphorylation between control and Aβ-treated cells was observed (**Figure 4G** and Supplementary Figure 1B).

### First Responses to Aβ42 Exposure Are Oxidative Stress and Subtle Nucleolar Stress

**FIGURE 5:**
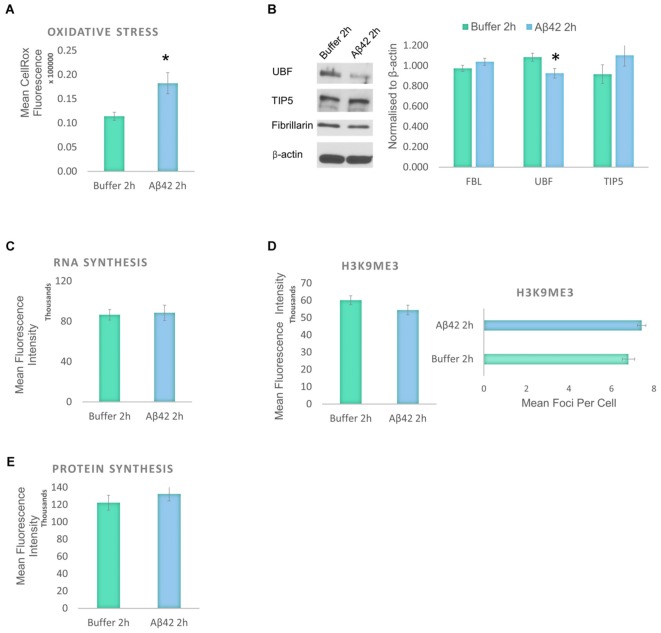
Early responses to Aβ42 exposure are oxidative stress and subtle nucleolar stress. **(A)** Flow Cytometry Experiment with CellROX Green showed that Aβ incubation for 2 h induces a significant level of oxidative stress. **(B)** Western blotting revealed that at the 2 h time point, Aβ causes a reduction of only UBF, not FBL or TIP5. Normalized to β-actin. Quantitative Click-iT RNA immunofluorescence labeling showed no change in newly synthesised RNA **(C)**; H3K9me3 **(D)**; or in newly synthesized proteins **(E)**. *N* = 5, ^∗^*P* < 0.05 (See also Supplementary Figure 4).

Aβ induces oxidative stress and a reduction in the levels of protein synthesis without causing significant DNA damage or affecting cell viability. To identify the earliest event induced by the Aβ incubation, we studied the changes that result from a short exposure to Aβ42 (**Figure 5**). D.SHSY5Y cells treated with Aβ42 oligomers for 2 h showed no impact on tau modifications and did not result in loss of cell viability or DNA damage (data not shown). However, Aβ42 incubation resulted in a significant increase in oxidative stress as measured using CellROX (**Figure 5A**), slightly lower than after 24 h incubation (**Figure 1C**). To examine whether this results in nucleolar stress, levels of FBL, UBF, and TIP5 were measured using western blotting of the whole cell fraction. Notably, while FBL and TIP5 remain unchanged, the short exposure to Aβ led to a modest, but significant reduction in UBF (**Figure 5B** and Supplementary Figure S3). Considering the critical role of UBF in rDNA transcription, this decrease may lead to a reduction of rDNA transcription in response to cellular stress (Boulon et al., 2010). However, the short Aβ exposure did not affect the levels of global RNA synthesis, H3K9me3 or protein synthesis (**Figures 5C–E**).

Hence, the findings from the exposure to Aβ for 2 and 24 h reveal that early consequences of Aβ incubation in the D.SHSY5Y are oxidative stress and subtle nucleolar stress. These become exacerbated over time, into a robust nucleolar stress with nucleolar tau redistribution that negatively impacts on the levels of RNA and protein synthesis within the cells.

## Discussion

In AD, changes in Aβ42 and tau levels appear decades before the onset of dementia (Jack et al., 2013). Several studies have revealed that alteration in the protein synthesis machinery, from the nucleolus to the ribosomes, occurs in the early stage of the disease (Ding et al., 2005; Hernandez-Ortega et al., 2015). Aβ is thought to cause subtle changes in neuronal function that gradually leads to cell death, whilst tau plays a very significant role in the process of neuronal dysfunction. Here, we investigated the early effects of oligomeric Aβ42 on the protein synthesis machinery implicated in the disease and examine the influence of Aβ42 on different species of nuclear tau and their distribution using differentiated human neuroblastoma cell line which expresses normal levels of human tau. Consistent with previous findings from human cortical slices treated with Aβ oligomers for 24 h (Sebollela et al., 2012), we found that the incubation of D.SHSY5Y with freshly prepared 10 μM Aβ42 oligomers for 24 h did not affect their viability or result in DNA damage. Previous studies have shown that Aβ42 treatment increases the level of reactive oxygen species (ROS) in primary neurons (De Felice et al., 2007). Here we revealed that Aβ42 induced oxidative stress, without exerting significant cell viability loss or DNA damage on these differentiated cells. Indeed, several lines of evidence have indicated that Aβ42 can induce oxidative stress, which is thought to play a critical role in AD progression (Butterfield et al., 2013).

Aβ toxicity is widely believed to aberrantly impact on tau protein, and several studies suggest that tau modifications, such as phosphorylation/dephosphorylation, may be a signature of general cellular stress (Egaña et al., 2003; Galas et al., 2006; Kátai et al., 2016). Here we reveal that Aβ42 incubation results in decreased levels of both nP-Tau and P-Tau, implying a relative increase in tau phosphorylated on Ser 195, 198, 199, and 202 and a decrease in tau Thr231 phosphorylation. Consistent with this, it has previously been shown that incubation of primary neurons with Aβ42, for up to 8 h, leads to a Pin1-mediated dephosphorylation of tau on Thr231, Ser199, Ser396, Ser400, and Ser404, with a progressive increase in its phosphorylation on Ser262 (Bulbarelli et al., 2009). This has been suggested to serve as an early response to prevent Aβ-induced tau hyperphosphorylation (Bulbarelli et al., 2009), which can be influenced critically by its phosphorylation at Thr231 (Lin et al., 2007). Furthermore, a recent study revealed that Aβ induces the phosphorylation of tau on Threonine 205 as an early mechanism for neuroprotection against excitotoxicity (Ittner et al., 2016). Thus, it seems that different stress signals or kinases could change the cellular activity and behavior of the tau molecule (Pooler et al., 2012; Lu et al., 2013b). Although recent studies have begun to shed light on how cellular stress impacts on nuclear tau (Sultan et al., 2011; Lu et al., 2013a,b; Noel et al., 2016; Maina et al., 2018), how this is affected in AD is not clear. Interestingly, it was recently shown that nuclear tau decreases in both the CA1 and dentate gyrus regions of the AD brain with disease progression (Hernandez-Ortega et al., 2015).

Cellular stress induces the cytoplasmic and nucleoplasmic distribution of nucleolar proteins (Kodiha et al., 2011). Here we revealed that Aβ42 incubation resulted in a reduction in nucleolar nP-Tau levels colocalizing with the nucleolar marker FBL. Furthermore, we observed a decrease in the levels of TIP5 and UBF as well as a decrease in rDNA transcription pointing to an increased level of nucleolar stress following Aβ42 treatment. Interestingly, oxidative and nucleolar stress appear to be earliest changes due to the Aβ toxicity, indicating the importance of the maintenance of cellular redox homeostasis and nucleolar integrity for cellular health. Human postmortem studies on the AD brain revealed that the nucleolus becomes affected in the early stage of the disease, which progresses with disease severity. UBF was shown to specifically reduce in both the CA1 and dentate gyrus from Braak stage 1 to 6 (Hernandez-Ortega et al., 2015). A similar altered level of nucleolar protein was also found in the Parkinson’s disease brain (Garcia-Esparcia et al., 2015). Collectively, our findings point to a potential early mechanism of Aβ toxicity, resulting in oxidative and nucleolar stress in the absence of cell death, and alteration in the phosphorylation level of tau epitopes, resulting in a change in tau’s localization within the nucleolus.

To investigate whether these changes affect the rRNA and protein levels, we explored the effect of Aβ on the protein translation machinery since rRNA are required for the assembly of functional ribosomes. A significant reduction in 45S-pre-rRNA, 18S, and 28S rRNA, and a subsequent decrease in global RNA synthesis indicates a global rearrangement of chromatin configuration, which may indicate a decrease in transcription. Indeed, this was associated with an increase in the heterochromatin marker, H3K9me3. AD-associated reduction in RNA and increase in heterochromatin formation has been reported in cortical neurons (Crapper et al., 1979; Mann et al., 1980; McLachlan et al., 1991). Consistent with this, microarray analysis of human cortical neurons challenged with Aβ oligomers previously showed that they result in ∼70% down-regulation of gene expression of 345 genes (Sebollela et al., 2012). Synthesized RNAs are translated into proteins through the recruitment and assembly of many factors, such as ribosomes. Depending on the metabolic activity of cells, rDNA transcription in mammalian cells accounts for ∼35 to 65% of total cellular transcription (Strohner et al., 2004). This reduction in protein synthesis could be due to a collective low availability of RNA and rRNA.

It has been observed that during stress, protein synthesis can also be inhibited through Serine 51 phosphorylation of α subunit of eukaryotic initiation factor 2 (eIF2α) (Holcik and Sonenberg, 2005). This is thought to enable the reduction of cellular energy expenditure and the production of unwanted proteins that could interfere with the stress response. In our model of Aβ-induced pathogenesis, nucleolar stress associated with a deficit in rDNA transcription appear to occur earlier than eIF2α phosphorylation. The deficits in protein synthesis observed in patients with MCI and early AD could be due to deficiencies in rRNA abundance, rather than eIF2α phosphorylation, since the reduction in rRNA, would translate to a decrease in the abundance of ribosomes and thus drop in protein translation. In our model, this links Aβ to the early changes in protein synthesis machinery in AD (Ding et al., 2005, 2006; Hernandez-Ortega et al., 2015). Consistent with early studies on chromatin and RNA changes in AD (Mann et al., 1980; McLachlan et al., 1991) and recent findings with Aβ (Sebollela et al., 2012), our results also indicate that the increased heterochromatin formation and reduction in the transcripts could contribute to the decrease in synthesized proteins due to the presence of Aβ oligomers. Although recent evidence on gene expression in AD shows variability between brain regions and proteins, AD pathology has been associated with differential changes in gene expression, where some pathways show decreased gene expression, while others show an increase (Liang et al., 2008a; Sebollela et al., 2012). Data from laser-capture micro-dissected neurons previously showed that some of the regions affected early in AD show under-expression of genes involved in energy metabolism (Liang et al., 2008b). Therefore, the decrease in RNA transcripts observed here reiterates the importance of Aβ in the early process of the disease.

## Conclusion

The amyloid cascade hypothesis places Aβ as the primary culprit for the pathogenesis of AD (Hardy and Higgins, 1992). Our findings here are consistent with the previous investigation showing a suppressive effect of Aβ42 on the cholinergic system, and gene expression (Sebollela et al., 2012), and supports a role for Aβ in influencing ribosome and protein synthesis deficits observed in this disease (Ding et al., 2005, 2006; Hernandez-Ortega et al., 2015). The changes are observed in the absence of overt toxicity (e.g., loss of cell viability) and correlate well with deficits in protein synthesis machinery that have been observed in MCI, a time-point in the progression of AD when there is no evidence of significant neuronal loss (Ding et al., 2005, 2006). Here we directly link Aβ in altered protein synthesis machinery to early cellular changes that impact on the progression of the disease, decades before full-blown AD (Jack et al., 2013). The results suggest that Aβ may play a role in driving deficits in the component of the translation machinery, from the nucleolus to the ribosomes, that occurs with AD progression (Hernandez-Ortega et al., 2015), perhaps an influence on nuclear tau which is essential for chromatin stability (Mansuroglu et al., 2016). These findings implicate Aβ as a culprit for the heterochromatinization and decrease in RNA levels that are reported to occur during the progression of this disease (Crapper et al., 1979; Mann et al., 1980; McLachlan et al., 1991).

## Availability of Data and Material

The datasets supporting the conclusions of this article are included within the article and its supporting information.

## Author Contributions

MM designed and conducted the experiments and analysis. LB and AD contributed significantly to experimental design and edited the paper. MM and LS wrote the paper. LS managed the research project with help from AD. All authors read and approved the final manuscript.

## Conflict of Interest Statement

The authors declare that the research was conducted in the absence of any commercial or financial relationships that could be construed as a potential conflict of interest.
