# Supplementary material for: The Involvement of Aβ42 and Tau in Nucleolar and Protein Synthesis Machinery Dysfunction
Source: Front Cell Neurosci. 2018 Aug 3;12:220. doi: 10.3389/fncel.2018.00220 (PMC6086011; doi:10.3389/fncel.2018.00220)
Supplement: Supplementary file 1 [file Image_1.PDF]

The involvement of Aβ42 and tau in nucleolar and protein synthesis machinery dysfunction

Mahmoud. B. Maina[1,2], Laura. J. Bailey[3], Aidan. J Doherty [3] & Louise. C. Serpell[1]

Supplementary Figure 1.

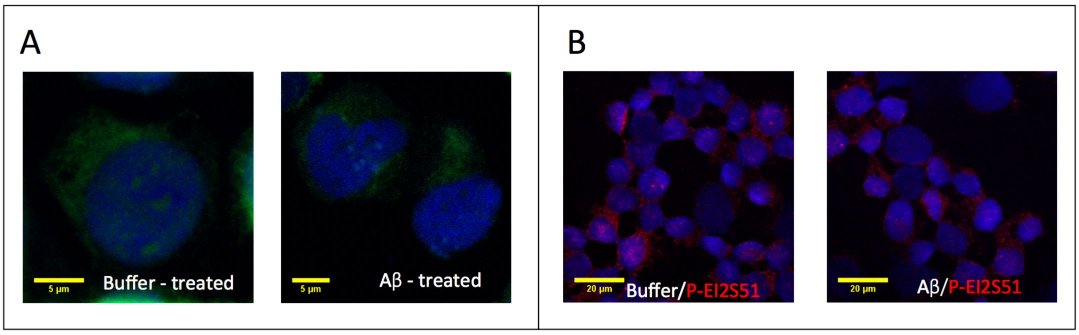

Supplementary Figure 1. (A) Quantitative Click-iT RNA immunofluorescence labelling showed that the Aβ causes a global reduction in newly synthesised RNA. (B) Quantitative immunofluorescence labelling for phosphor S51 eukaryotic translation initiation factor 2A (EIF2-P) showed no changes following the Aβ treatment. N=3

Supplementary figures below show the full blots that are shown in Figures 2-4 in the main text.

Supplementary Figure 2.

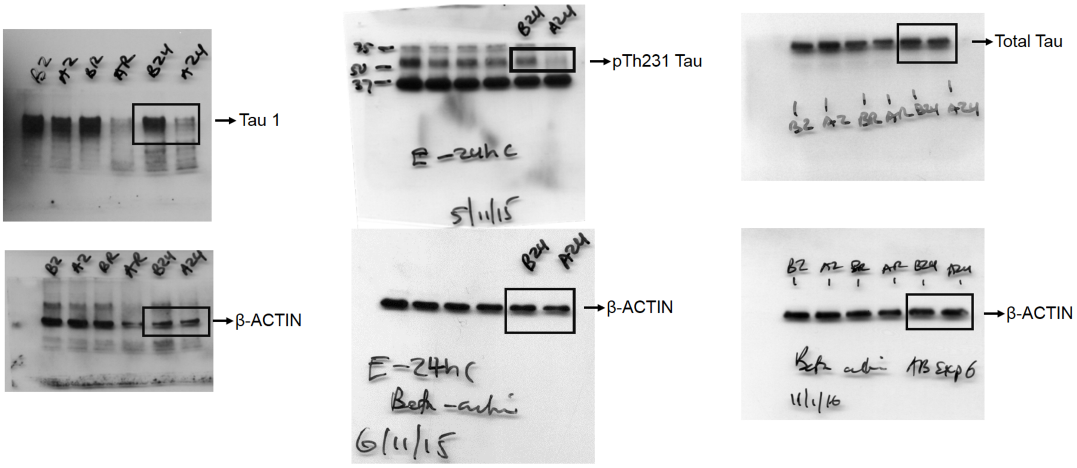

Supplementary Figure 2. Aβ42 oligomers change the phosphorylation of tau epitopes and alter nuclear tau localisation 24h post-incubation. Western blotting on whole cell extracts showing the levels of Tau Thr231 (P-Tau), Tau-1 (nP-Tau) and total tau (T-Tau) following Aβ administration. Normalised to β-actin. Key; B24 for Buffer-treated cells for 24h and Aβ24h for Aβ42-treated cells for 24h. **Boxed bands represent bands used in the main article in Figure 2A.**

Supplementary Figure 3.

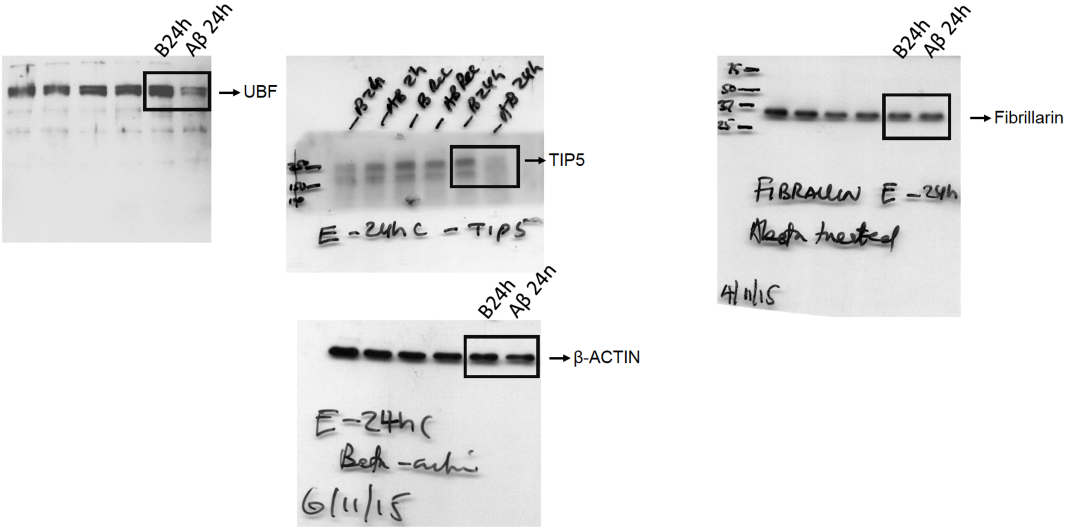

Supplementary Figure 3. Aβ42 induces nucleolar stress and inhibit RNA and Protein Synthesis 24h post-incubation. Western blotting revealed that Aβ treatment led to a significant decrease in UBF, TIP5, but not FBL. Normalised to β-actin. Key; B24 for Buffer-treated cells for 24h and Aβ24h for Aβ42-treated cells for 24h. **Boxed bands represent bands used in the main article in Figure 3A.**

Supplementary Figure 4.

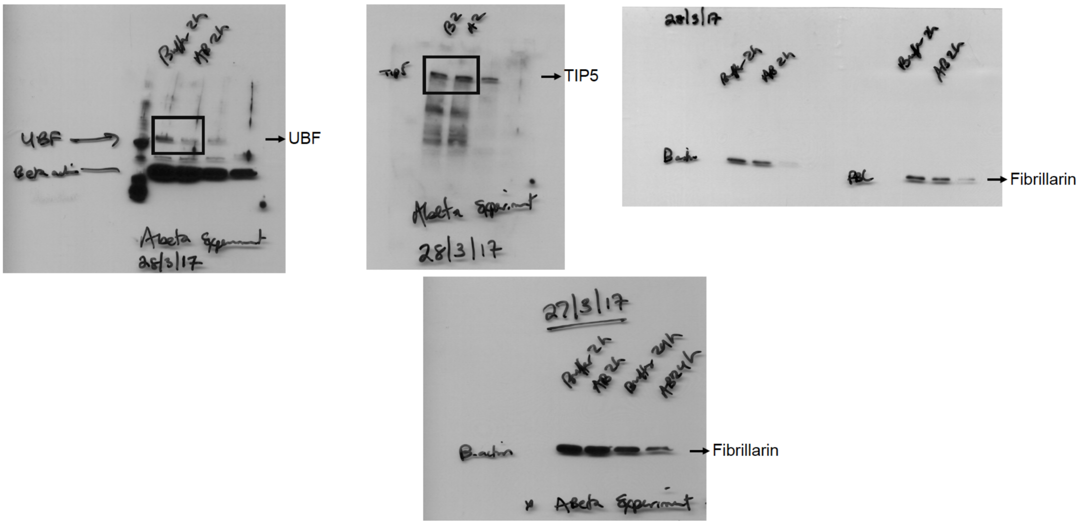

Supplementary Figure 4. Early responses to Aβ42 exposure are oxidative stress and subtle nucleolar stress. Western blotting revealed that at the 2h time point, Aβ causes a reduction of only UBF, not FBL or TIP5. Normalised to β-actin. Key; B2 for Buffer-treated cells for 2h and Aβ2h for Aβ42-treated cells for 2h. **Boxed bands represent bands used in the main article in Figure 4B.**
