# Supplementary material for: The Involvement of Aβ42 and Tau in Nucleolar and Protein Synthesis Machinery Dysfunction
Source: Front Cell Neurosci. 2018 Aug 3;12:220. doi: 10.3389/fncel.2018.00220 (PMC6086011; doi:10.3389/fncel.2018.00220)
Supplement: Supplementary file 2 [file Table_1.doc]

**Supplementary Table 1.** Antibodies

| **Name and Catalogue number** | **Dilution** | **Supplier** |
| --- | --- | --- |
| Rabbit polyclonal anti-TAU antibody (SAB4501831) | IF: 1/100 WB: 1:1000 | Sigma-Aldrich |
| Mouse-Phosphor-Tau (Thr231) Antibody (MN1040) | IF: 1/50 | Thermo Fisher Scientific |
| Mouse Anti-Tau-1 antibody (MAB3420) | IF: 1/200, WB: 1/1000 | Millipore |
| Mouse Anti-gamma H2A.X antibody (phospho S139) antibody [9F3] | IF: 1/500 | Abcam |
| Rabbit Anti-Fibrillarin antibody (ab5821) | IF:1/200, WB:1/1000 | Abcam |
| Rabbit Anti-trimethyl-Histone H3 (Lys9) antibody (07-442) | IF:1/200 | Millipore |
| Rabbit Anti-UBF antibody (H-300) sc-9131 | IF:1/200; WB:1/1000 | Santa Cruz Biotechnology, Inc |
| Rabbit Anti-TIP5 Polyclonal Antibody (49-1037) | WB:1/200, | Life technologies |
| Rabbit- Anti-EIF2S1 (phospho S51) antibody [E90] (ab32157) | IF: 1/200 | Abcam |
| Mouse Monoclonal Anti-β-Actin antibody (**A5316**) | WB:1/5000 | Sigma-Aldrich |
| Alexa Fluor® 555 Goat Anti-Mouse IgG (H+L) (A31622) | 1/500 | Invitogen |
| Alexa Fluor® 555 Goat Anti-Rabbit IgG (H+L) (A31630) | 1/500 | Invitogen |
| Alexa Fluor® 488 Goat Anti-Mouse IgG (H+L) | 1/500 | Invitogen |
| Alexa Fluor® 488 Goat Anti-Rabbit IgG (H+L) (A31628) | 1/500 | Invitogen |
| Goat Anti-Rabbit IgG H&L (HRP) (ab6721) | 1/5000 | Abcam |
| Anti-mouse IgG, HRP-linked Antibody (7076) | 1/1000 | Cell Signalling |

Key: IF – immunofluorescence, WB – Western blotting
