# Supplementary material for: The Involvement of Aβ42 and Tau in Nucleolar and Protein Synthesis Machinery Dysfunction
Source: Front Cell Neurosci. 2018 Aug 3;12:220. doi: 10.3389/fncel.2018.00220 (PMC6086011; doi:10.3389/fncel.2018.00220)
Supplement: Supplementary file 3 [file Table_2.docx]

**Supplementary Table 2: List of primers used for qPCR**

| **Primer name** | **Forward sequence** | **Reverse sequence** |
| --- | --- | --- |
| TIP5 | Taqman Assay (Lifetechnologies) | assay ID; Hs00203782_m1 |
| Fibrillarin | TaqMan Assay (Life technologies) | assay ID; Hs01070449_m1 |
| UBF | Taqman Assay (Lifetechnologies) | assay ID; Hs00610730_g1 |
| RNA18S5 | TaqMan Assay (Life technologies) | assay ID; Hs03928985_g1 |
| RNA28S5 | Taqman Assay (Lifetechnologies) | assay ID; Hs03654441_s1 |
| TBP | Taqman Assay (Lifetechnologies) | assay ID; Hs00427620_m1 |
| Β-actin (ACTB) | Taqman Assay (Lifetechnologies) | assay ID; Hs01060665_g1 |

| Name | Forward | Reverse | probe |
| --- | --- | --- | --- |
| RNA45S | CACCCTCGGTGAGAAAAG | CTACCATAACGGAGGCAG | CTTCTCTAGCGATCTGAGAGGCGTGCC |
